# Daily blue-light exposure shortens lifespan and causes brain neurodegeneration in *Drosophila*

**DOI:** 10.1038/s41514-019-0038-6

**Published:** 2019-10-17

**Authors:** Trevor R. Nash, Eileen S. Chow, Alexander D. Law, Samuel D. Fu, Elzbieta Fuszara, Aleksandra Bilska, Piotr Bebas, Doris Kretzschmar, Jadwiga M. Giebultowicz

**Affiliations:** 1 Department of Integrative Biology, Oregon State University, Corvallis, OR 97331 USA; 2 Oregon Institute of Occupational Health Sciences, Oregon Health and Science University, Portland, OR 97239 USA; 3 Department of Animal Physiology, Faculty of Biology, University of Warsaw, 02-096 Warsaw, Poland

**Keywords:** Genetics, Ageing

## Abstract

Light is necessary for life, but prolonged exposure to artificial light is a matter of increasing health concern. Humans are exposed to increased amounts of light in the blue spectrum produced by light-emitting diodes (LEDs), which can interfere with normal sleep cycles. The LED technologies are relatively new; therefore, the long-term effects of exposure to blue light across the lifespan are not understood. We investigated the effects of light in the model organism, *Drosophila melanogaster*, and determined that flies maintained in daily cycles of 12-h blue LED and 12-h darkness had significantly reduced longevity compared with flies maintained in constant darkness or in white light with blue wavelengths blocked. Exposure of adult flies to 12 h of blue light per day accelerated aging phenotypes causing damage to retinal cells, brain neurodegeneration, and impaired locomotion. We report that brain damage and locomotor impairments do not depend on the degeneration in the retina, as these phenotypes were evident under blue light in flies with genetically ablated eyes. Blue light induces expression of stress-responsive genes in old flies but not in young, suggesting that cumulative light exposure acts as a stressor during aging. We also determined that several known blue-light-sensitive proteins are not acting in pathways mediating detrimental light effects. Our study reveals the unexpected effects of blue light on fly brain and establishes *Drosophila* as a model in which to investigate long-term effects of blue light at the cellular and organismal level.

## Introduction

Natural light is essential for the entrainment of circadian clocks, which leads to temporal coordination of physiology and behavior. However, emerging evidence suggests that increased exposure to artificial light is a risk factor for sleep and circadian disorders.^1,2^ With the prevalent use of LED lighting and device displays, humans are subjected to increasing amounts of light in the blue spectrum since commonly used LEDs emit a high fraction of blue light, often peaking at 460 nm (these lights appear white due to the addition of broad-spectrum yellow garnet phosphor).^3^ Blue light may affect human eyes,^4^ and recent data suggest that extraocular light may impact human brain physiology.^5^ However, the consequences of daily exposure to blue-enriched light across the lifespan are not known.^6^

Research on model organisms suggests that visible light may have a range of detrimental effects. A single acute blue-light exposure causes photoreceptor death in the retina of mice and flies.^7–9^ There are reports that light may shorten longevity in *Drosophila*,^10–12^ but the mechanisms underlying the effects of light on lifespan have not been pursued. Here, we characterized the effects of different light exposures on the mortality and aging phenotypes in *Drosophila*. We report that adult flies maintained in cycles of 12h light and 12h darkness show symptoms of accelerated aging, such as impaired locomotor performance, brain neurodegeneration, and reduced lifespan compared with flies reared in constant darkness. We identified blue light as responsible for these aging phenotypes and investigated the involvement of light-responsive pathways in mediating the detrimental effects of blue light on the brain.

## Results

### Blue light and aging

**Fig. 1:**
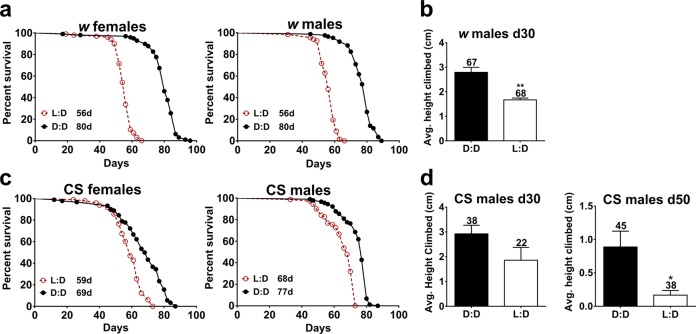
White fluorescent light shortens fly lifespan and decreases mobility. **a** Adult *white* (*w*) flies aged in constant darkness (D:D) have a significantly extended lifespan compared with those aged in white fluorescent light (L:D) (Log-rank test, *p* < 0.0001). **b** Average climbing ability was significantly lower in 30-day-old *w* males kept in L:D versus those kept in D:D (unpaired *t* test, *p* = 0.0066). **c** Canton S (CS) adult flies aged in D:D have a significantly extended lifespan compared with those aged in L:D (Log-rank test, *p* < 0.0001). **d** Average climbing ability was lower but not significant in 30-day-old CS males kept in L:D versus those kept in D:D, and significantly lower in 50-day-old CS males kept in L:D versus those kept in D:D (unpaired *t* test, *p* = 0.0434). For longevity experiments in (**a**, **c**), *N* = 100 for each genotype and light condition. Numbers above bars indicate the sample size in each light condition. Error bars show standard error of the mean (SEM)

To investigate whether light affects *Drosophila* longevity, we first compared the lifespan of *white* (*w*^*1118*^, hereafter *w*) adult flies kept in daily cycles of 12-h white fluorescent light alternating with 12 h of darkness (L:D) or in constant darkness (D:D). Survival of flies in D:D was significantly extended compared with those in L:D (Log-rank test, *p* < 0.0001) and their median lifespan was extended by 42% (Fig. 1a). The difference in mortality could be caused by delayed aging or by other factors. Aging in flies is associated with slower climbing up the vial walls, which can be measured by using the rapid iterative negative geotaxis (RING) assay.^13^ To determine whether the lifespan extension of D:D flies was associated with delayed aging, we measured vertical locomotion by RING. Middle-aged (30-day-old) males kept in D:D had significantly better average climbing ability than flies kept in L:D (Fig. 1b), suggesting that reduced lifespan of L:D flies may be due to accelerated aging. The lack of pigment granules in the retina makes *w* flies sensitized to light;^14^ therefore, we also tested whether the longevity of wild-type Canton S (CS) flies was affected by light. Indeed, the lifespan of CS males and females was significantly reduced in L:D compared with D:D (Log-rank test, *p* < 0.0001), albeit not as strongly as in *w* flies (Fig. 1c). Consistent with these results, 30-day-old CS flies in L:D showed a trend toward reduced average climbing ability, which became statistically significant at a later age of 50 days (Fig. 1d).

**Fig. 2:**
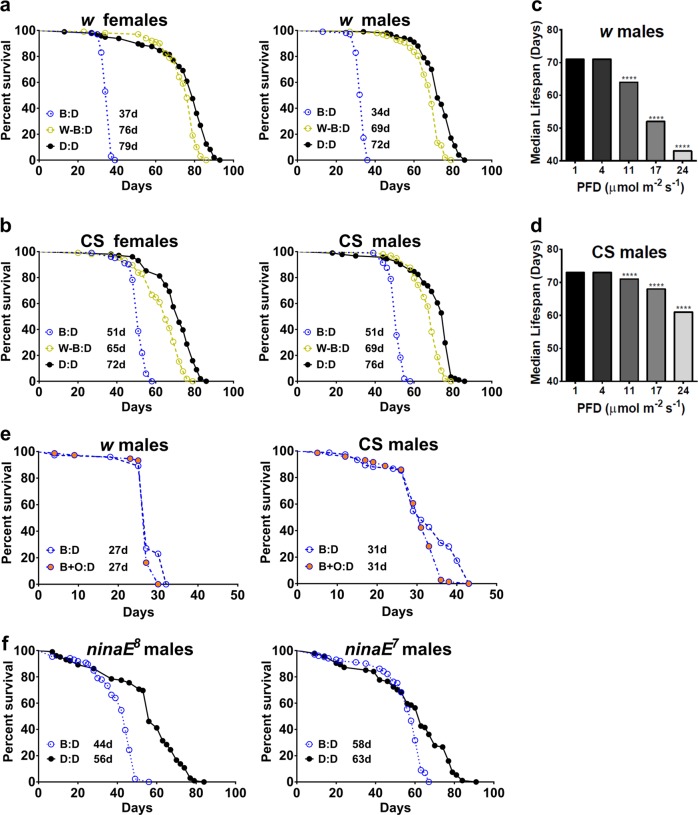
Light in the blue spectrum is responsible for the decrease in fly lifespan. Lifespan of *w* **a** and CS **b** flies is dramatically reduced in B:D compared with D:D (Log-rank test, *p* < 0.0001), but minimally reduced in flies aged under white LEDs lacking blue wavelengths by means of a yellow filter (W–B:D). Median lifespan of *w* **c** and CS **d** flies in B**:**D is reduced with increasing photon flux density (PFD). Statistics shown are from pairwise comparisons of the corresponding mortality curves that showed a dose-dependent effect, increasing PFD from 4 to 11, from 11 to 17, and from 17 to 24 µmol each, causing a significant increase in mortality (Log-rank tests with Bonferroni multiple correction, *p* < 0.0001). Note that the y axis does not start at 0 to highlight these differences in median lifespan. **e** Survival of *w* and CS males kept in B:D or in B:D with added orange light (B + O:D). **f** Mortality curves of the white-eyed *ninaE*^*8*^ and red-eyed *ninaE*^*7*^ mutants in B:D and D:D. In all of the above experiments, *N* = 100 for each genotype and light condition

The spectral composition of light used in the above experiments showed a substantial blue component (Supplementary Fig. 1a); therefore, we tested the contribution of blue wavelengths commonly used in human environments (LED with peak wavelength at ~460 nm) to the lifespan reduction. Lifespan was measured in flies kept in daily cycles of 12-h blue LED light and 12 h of darkness (B:D), or white LED light with blue wavelengths blocked by a yellow filter (W–B:D) (Supplementary Fig. 1b). To equalize the amount of exposure across light sources, all light sources hereafter were adjusted to emit similar photon flux density (PFD) as L:D, ranging from 20 to 30 µmol m^−2^ s^−1^, at the level where flies were kept. Compared with flies aged in D:D, the median lifespan of *w* flies was reduced by ~50% in B:D but only by 4% in W–B:D light (Fig. 2a). Likewise, blue light caused a more dramatic (~30%) reduction in the median lifespan of CS flies compared with W–B light, which shortened median lifespan by ~10% (Fig. 2b). We also determined that the lifespan reduction of both *w* and CS flies corresponded to increased intensity of blue light (Fig. 2c, d). Pairwise comparisons of mortality curves showed a dose-dependent effect, namely, increasing PFD from 4 to 11, from 11 to 17, and from 17 to 24 µmol each caused a significant increase in mortality (Log-rank tests with Bonferroni multiple correction, *p* < 0.0001). Taken together, these results suggest that irradiation by blue wavelengths is mainly responsible for the reduced longevity of flies exposed to light.

Blue light activates Rhodopsin 1, the prevalent opsin in the fly retina, which then requires exposure to orange light in order to regenerate.^15^ To test whether lack of orange light may contribute to the reduced lifespan, we kept flies under B:D alone or B:D of similar intensity with the addition of orange LED light (peak at 600 nm, 1.5 µmol m^−2^ s^−1^) to allow for Rhodopsin regeneration. Median lifespan of both *w* and CS flies was not extended by the addition of orange light (Fig. 2e), suggesting that defects in rhodopsin processing are not responsible for the reduced lifespan of flies maintained in blue light. It was reported that blue-light-induced photoreceptor death is ameliorated by mutations in the gene encoding Rhodopsin 1 (*ninaE*), which disrupt phototransduction;^9^ therefore, we tested the effects of blue light on the lifespan of *ninaE*^7^ and *ninaE*^8^ mutants, both with reduced rhodopsin levels.^9,16^ The lifespan of white-eyed *ninaE*^8^ flies was shortened significantly in B:D compared with D:D (Log-rank test, *p* < 0.0001) with median lifespan reduced by 21% (Fig. 2f). The lifespan of red-eyed *ninaE*^7^ flies was also shortened significantly in B:D compared with D:D (Log-rank test, *p* < 0.0001), with median lifespan reduced by 9% (Fig. 2f). The fact that the magnitude of lifespan reduction was smaller in mutants with impaired phototransduction than in *w* or CS flies suggests that phototransduction may partially contribute to the detrimental effects of blue light.

Blue light acts in the entrainment of the circadian clock even at low intensities;^17^ however, we reasoned that levels of blue light that negatively affect longevity could have damaging effects on the clock. To test this, we recorded locomotor activity of flies held in L:D or B:D cycles for 5 days and then transferred to D:D for 5 days. Flies in both L:D and B:D showed prominent morning and evening activity peaks; however, B:D flies were more active throughout the entire light phase, especially at younger ages (Supplementary Fig. 2). Upon transfer to D:D, young flies from both regimes showed strong free-running circadian rhythms (Supplementary Fig. 2), suggesting that light used in this study is not damaging to the clock. Given these results, we then tested whether disruption of the circadian clock increases the susceptibility to blue light, as it is known that an intact clock confers resistance to many stresses.^18,19^ We determined that the lifespan of flies with disrupted clocks due to a mutation in the core clock gene *period* (*per*^*01*^) was not reduced in B:D compared with *w* control flies with an intact clock (Supplementary Fig. 3), suggesting that a functional clock is not protective against the blue-light exposure used in our experiments.

**Fig. 3:**
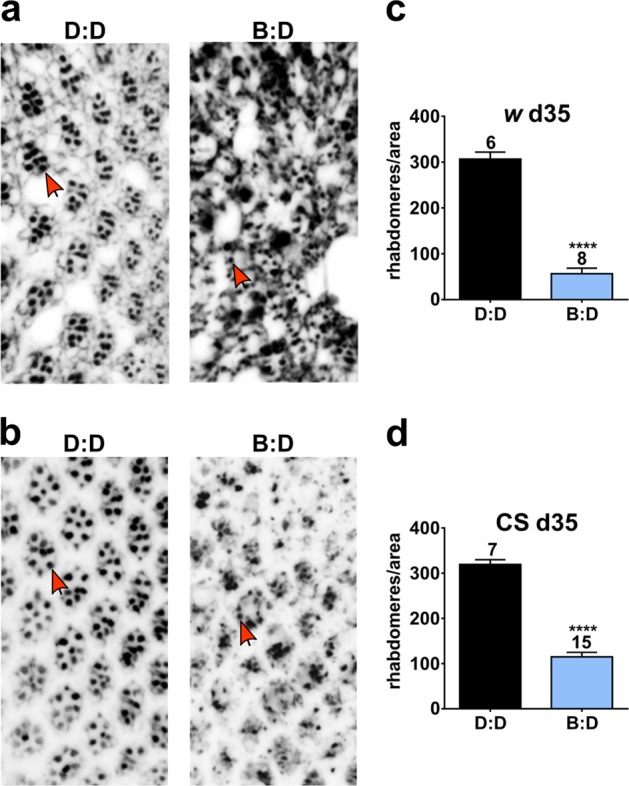
Retinal photoreceptors degenerate under blue light in flies with white or red eyes. Representative retinal cross sections of 35-day-old *w* **a** and CS **b** males in D:D and B:D. Red arrows point to identifiable rhabdomeres. **c**, **d** The average number of rhabdomeres is significantly reduced in 35-day-old *w* and CS males in B:D compared with D:D (unpaired *t* test, ****p < 0.0001). Numbers above bars indicate the sample size in each light condition. Error bars show SEM

It has been reported that mammalian and fly retinal photoreceptor cells subjected to acute strong blue light become damaged;^9,20^ therefore, we asked whether photoreceptor cells are affected by daily 12-h exposure to moderate blue light. The fly retina consists of ~800 identical units called ommatidia, containing 6 outer and 2 inner photoreceptor cells (PR), each possessing a rhabdomere consisting of tightly packed microvilli where the phototransduction occurs. We examined histologically the health of the PRs in *w* and CS flies kept in D:D or B:D by counting the number of identifiable rhabdomeres (arrows, Fig. 3) on the same area of retinal cross sections in different conditions. At the age of 35 days, *w* and CS flies in D:D showed the regular arrangement of PRs with the dark rhabdomeres clearly distinguishable (Fig. 3a, b). In contrast, retinal degeneration and disorganized rhabdomeres were evident at this age in flies kept in B:D (Fig. 3a, b). A quantification confirmed a significant reduction in the average number of distinct rhabdomeres in both *w* and CS flies in B:D relative to D:D (Fig. 3c, d). By comparing rhabdomere loss between genotypes in B:D, we determined that it was more significant (unpaired *t* test, *p* = 0.0018) in *w* flies than in CS flies with normal eye pigmentation. This is consistent with higher PR degeneration reported previously in *w* flies in unspecified light conditions.^21^

**Fig. 4:**
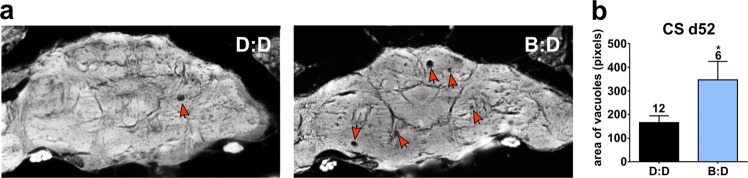
Blue-light exposure leads to neurodegeneration in the aging fly brain. **a** Representative brain sections showing brain vacuoles (red arrows) in 52-day-old CS males in D:D compared with B:D. **b** Average area of vacuoles is significantly higher in B:D (unpaired *t* test, *p* = 0.0165). Numbers above bars indicate the sample size in each light condition. Error bars show SEM

Since PR damage occurred even in wild-type flies with normal eye pigmentation, we next asked whether deeper brain tissues are affected by blue-light exposure. To examine the central brain, heads of CS flies aged in D:D or B:D for 52 days were sectioned to measure the size of vacuoles indicative of neuronal loss. A significant increase in the average area of brain vacuolization was detected in CS flies in B:D compared with age-matched flies in D:D (Fig. 4).

**Fig. 5:**
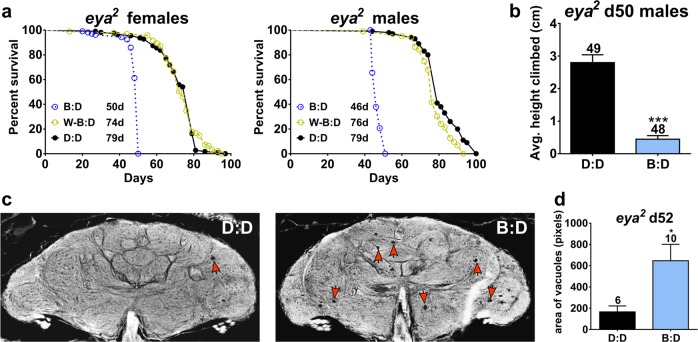
Flies lacking retina show reduced lifespan and brain neurodegeneration in blue light. **a** Lifespan of *eyes-absent* mutant (*eya*^2^) flies is significantly reduced in B:D compared with D:D (Log-rank test, *p* < 0.0001 for males and females), but is similar in W–B:D conditions (*N* = 100 for each light condition). **b** Aged *eya*^2^ males show a significant reduction in the average vertical climbing ability in B:D compared with D:D (unpaired *t* test, *p* = 0.0009). **c** Representative brain sections showing brain vacuoles (red arrowheads) in 52-day-old *eya*^2^ males in D:D and B:D. **d** The average area of brain vacuolization of 52-day-old *eya*^2^ males was significantly increased in B:D compared with D:D (unpaired *t* test, *p* = 0.0352). Numbers above bars indicate the sample size in each light condition. Error bars show SEM

The observation that blue-light exposure leads to damage in both PR and the brain raised the question of whether PR degeneration is causally involved in brain neurodegeneration, or alternatively, whether blue light affects the brain independent of the retinal status. To address this, we used *eyes-absent* (*eya*^2^) mutants,^22^ which do not develop compound eyes and thus lack PRs. The lifespan of *eya*^2^ flies was significantly shortened in B:D compared with D:D (Log-rank test, *p* < 0.0001), with median lifespan reduced by 37% for males and 42% for females (Fig. 5a). In contrast, median lifespan was reduced by only 6% and 4%, respectively, in males and females kept in white light with blue wavelengths blocked (W–B:D) compared with flies kept in D:D (Fig. 5a). Climbing ability was also severely compromised in *eya*^2^ flies in B:D compared with D:D (Fig. 5b). As in flies with normal eyes, this behavioral deficit was associated with a significant degree of brain degeneration, measured as an increased area of vacuoles in B:D *eya*^2^ flies (Fig. 5c, d). In an additional experiment, we measured the lifespan of another mutant lacking PRs, *sine oculis* (*so*^1^), and found that their lifespan was also significantly shortened by blue light; the median lifespan of *so*^1^ in B:D was reduced by 19% compared with D:D (Supplementary Fig. 4). Together, these data suggest that accelerated mortality and locomotor impairments of flies maintained in B:D may occur independently of retinal damage. We hypothesize that brain neurodegeneration is a culprit in accelerating aging; however, other organs not studied here may be also involved.

To begin investigating molecular pathways mediating the damaging action of blue light on the brain, we first considered cryptochrome, the blue-light-sensitive photoreceptor protein encoded by the gene *cry*. In flies, the CRY protein is the major light sensor for the entrainment of the circadian clock,^23,24^ and it is involved in modulation of neuronal activity and behavior by blue light.^25,26^ To test whether CRY could mediate the phototoxicity of blue light, we measured the lifespan of flies with genetically manipulated *cry* expression held in B:D or D:D. We found that neither a null mutation in the *cry* gene nor overexpression of *cry* affected survival in B:D conditions (relative to D:D) compared with their respective controls (Supplementary Fig. 5a), suggesting that CRY is not involved in the lifespan alterations caused by blue light. In addition to *cry*, we tested whether the recently identified Rhodopsin 7 (Rh7) plays a role in inducing the aging phenotypes. RH7 protein is sensitive to blue light and its mRNA is weakly expressed in both the brain and the retina.^27^ We determined that median lifespan was similarly shortened in B:D relative to D:D, both in *Rh7*^*1*^ mutants and in flies overexpressing *Rh7*, compared with their respective controls (Supplementary Fig. 5b), suggesting that this chromoprotein is not involved in mediating the effects of daily blue-light exposure on longevity. We note that it is still possible that removing all photoreceptive pathways (i.e., cry and rhodopsins together) could reduce blue-light-induced damage.

**Fig. 6:**
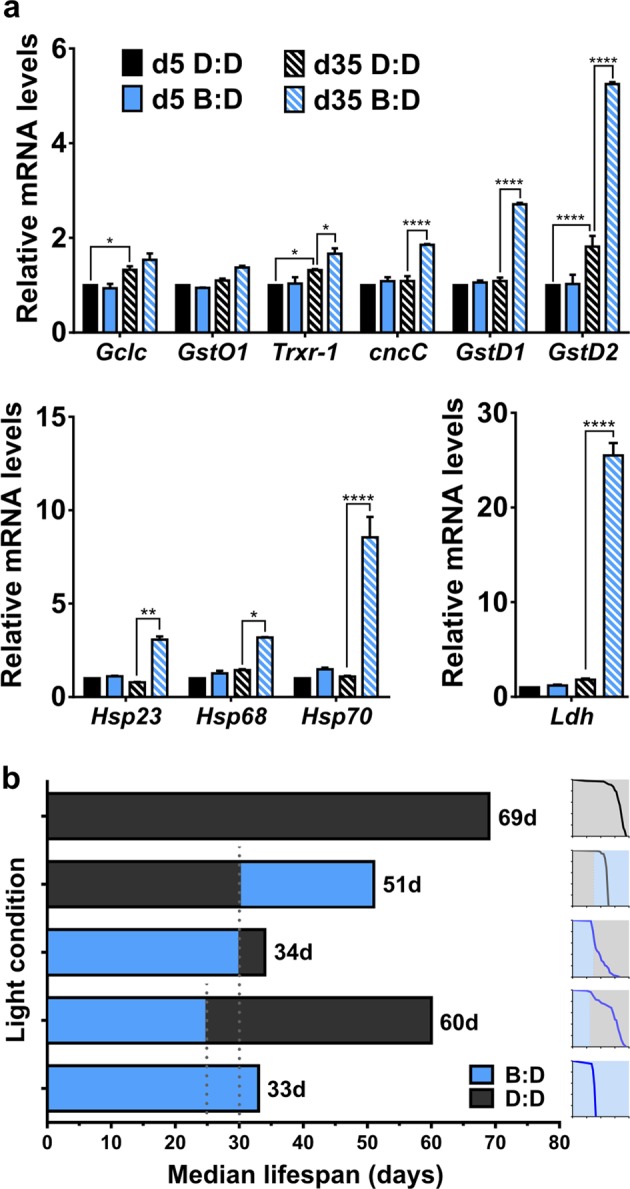
Flies maintained in blue light show induction of stress-response genes by day 35. **a** Expression levels of the indicated stress-response genes in heads of 5- and 35-day-old *w* males maintained in B:D or D:D. For each gene measured, values from qPCR are reported as fold change relative to expression in young flies in D:D set as 1. Statistics by 2-way ANOVA (****p < 0.0001; **p < 0.01; *p < 0.05). Bars show the average of two biorepeats; error bars show SEM. **b** Median lifespan of *w* flies exposed to B:D and/or D:D for the durations indicated by blue or black bars. Flies kept in B:D for the first 30 days of adulthood and then transferred to D:D had a similar median survival to those kept in B:D throughout their entire life, while flies kept in B:D for the first 25 days of adulthood were able to survive much longer (Log-rank test, *p* < 0.0001). Flies kept in D:D for the first 30 days of adulthood experienced an increased mortality rate upon transfer to B:D. Mortality curves for each condition are shown on the right

What are the proximate causes of premature aging of flies in B:D? Our recent RNA-seq study comparing the diurnal transcriptome in heads of young and old flies demonstrated that several stress-response genes are upregulated in heads of 55-day-old *w* flies kept in L:D 12:12 cycles, and their maximal expression over a 24-h period occurred after 12 h of light exposure.^28^ These genes also become induced in young flies kept in L:D but subjected to oxidative stress by treatment with 100% oxygen.^28^ Given our observation that L:D shortens the lifespan in flies (Fig. 1), and reports that blue light induces oxidative stress in retinal cells^9,20^ and in the nematode *Caenorhabditis elegans*,^29^ we tested whether blue light increased the expression of genes known to be induced by oxidative stress. The expression of selected stress-response genes was measured in heads of day 5 or day 35 *w* flies maintained in B:D and collected at the end of their daily 12 h of blue-light exposure. To discern the effects of light, we collected simultaneously 5- or 35-day-old *w* flies maintained in D:D; these flies are expected to show average expression of diurnal genes due to the absence of clock entrainment by light. Some of the known oxidative stress-response genes (*Gclc*, *GstO1*) were not upregulated in B:D; however, expression of several other genes was significantly increased in 35-day-old flies in B:D compared with age-matched D:D controls (Fig. 6a). These included *cnc* (the fly homolog of the transcription factor *Nrf2*), thioredoxin reductase *Trxr-1*, glutathione S transferases *GstD1* and *GstD2*, and several heat-shock proteins: *Hsp23*, *Hsp68*, and *Hsp70*. Most of the examined genes (with the exception of *Gclc*, *Trxr-1*, and *GstD2*) did not increase expression in 35-day-old D:D flies compared with 5-day-old D:D flies, suggesting that blue light plays a much bigger role in upregulation of stress-response genes than aging by itself. We also observed strong upregulation of the metabolic gene, lactate dehydrogenase (*Ldh*), which is known to increase with aging and stress.^28^ Importantly, none of the examined genes showed an increase in 5-day-old flies kept in B:D compared with D:D, suggesting that the cumulative action of blue light over many days is needed to induce stress-response genes, or that response to blue light is age-dependent (Fig. 6a). To explore these possibilities further, we tested survival of flies exposed to B:D or D:D for a set number of days and then switched to the opposite conditions. We kept *w* flies in B:D throughout their life, or for the first 25 or 30 days of adult life followed by a transfer to D:D, and compared their lifespan. As shown in Fig. 6b, exposure for the first 25 days of adulthood caused some flies to die within a few days, but most of the remaining flies survived nearly as long as flies that were always kept in D:D. However, exposing flies to B:D for the first 30 days of adulthood (only 5 days longer than in the previous experiment) followed by a transfer to D:D resulted in the majority of flies dying shortly after the switch to D:D. These flies had a median lifespan of 34 days, similar to the 33 days of controls kept continually in B:D. In a reverse experiment, we kept flies in D:D for 30 days and then exposed them to B:D for the rest of their lives. The median survival of these 30-day-old flies was 21 days after the switch to B:D, while the median lifespan of young flies exposed to B:D was 34 days. These results suggest two conclusions. First, blue light has cumulative damaging effects, but the damage can be halted upon removal of this type of stress, provided that it does not accumulate beyond a certain irreversible threshold that causes death. Second, blue-light damage affects flies differently across their lifespan with vulnerability to this part of the visible spectrum increasing with age. In other words, blue-light-induced damage seems to accumulate faster with advancing age.

## Discussion

Understanding the effects of blue light on various life processes is becoming an increasingly important health issue as humans are exposed to more blue-enriched LED illumination for most of the day, or even at night due to shift work and light pollution in large cities.^6^ However, long-term consequences of increased daily blue-light exposure across the human lifespan are not known. In this study, we demonstrate that daily exposure to 12 h of visible light in the blue part of the spectrum accelerates aging in *Drosophila*. Light causes not only retinal damage but also neurodegeneration in the central nervous system, which may be involved in the premature decline in climbing ability and early mortality. Our data also suggest that susceptibility to light increases with age and repetitive exposure to blue light induces the expression of stress-response genes.

The detrimental effects of light on longevity have been reported recently in *C. elegans*;^29^ exposing these nematodes to white light or different parts of the light spectrum significantly reduced their lifespan,^29^ suggesting a broad susceptibility to light in this species, albeit with stronger effects of shorter wavelengths. Our data suggest that blue light is driving the aging phenotypes in flies since it dramatically reduced the lifespan, while light in the 500–700-nm range with similar photon flux only minimally affected longevity compared with D:D.

Numerous studies reported that light in the blue spectrum causes damage to retinal cells in vitro and in vivo in mammals and *Drosophila*.^7,9,30^ While these studies employed acute strong light, we show that photoreceptor cells of aging flies degenerate in response to 12 h of daily exposure to moderate blue light. This degeneration was more pronounced in *w* flies than in age-matched CS flies, presumably due to a lack of the screening red pigment in the former genotype.^21^ Blue-light-induced degeneration of fly retinal photoreceptors appears to involve the phototransduction cascade, as retinal damage is mitigated by mutations that impair phototransduction.^9^ We show here that this may not be the case for organismal aging, because these mutations only partially rescue the lifespan reduction caused by blue light. Likewise, addition of orange light, which is known to deactivate rhodopsin, did not rescue the lifespan. Thus, it appears that the effects of light on retinal versus organismal aging may be mediated by different mechanisms.

A surprising outcome of our study is that blue light not only damaged the retina, but also caused neurodegeneration in the brain. A significant age-specific increase in the area of vacuoles indicative of neuronal death was observed in brains of flies in B:D compared with age-matched flies in D:D. In addition, we demonstrate that blue-light-induced damage to the brain occurs whether or not the retina is present, suggesting that light can affect the brain directly and independently of the visual system degeneration. To address possible blue-light-activated pathways in the brain, we tested the involvement of the photoreceptive proteins CRY and RH7, both of which are expressed in the brain, and determined that neither loss nor overexpression of either protein significantly affected fly survival in blue light. Further studies will be required to dissect the input pathways mediating the effects of blue light on the brain. We note that the effects of light on extra-retinal tissues may not be limited to invertebrates. There are reports that the exposure of rats or mice to constant light for several months was associated with a significant reduction in the number of dopaminergic neurons.^31,32^ In addition, transcranial blue light may impact human brain activity.^5^ Taken together, these data suggested that the question of possible detrimental effects of light on brain aging deserves more attention.

We hypothesize that light-induced brain neurodegeneration may be the main cause of the decreased vertical mobility and reduced lifespan. However, at this time, we cannot exclude the possibility that other fly tissues could be affected by blue light and contribute to the accelerated aging. For example, the study on *C. elegans* showed that mitochondria in the muscles were damaged by constant light exposure.^29^

Several studies determined that blue-light exposure results in the generation of reactive oxygen species (ROS) in the retina of mice^20^ and flies^9^ and even in skin cells.^33^ Light exposure that shortens the lifespan of *C. elegans* also increases ROS levels and induced an unfolded protein response.^29^ The expression of selected stress-response genes was induced by light in worms^29^ and in photoreceptor cells of the fly retina.^34^ Our data are consistent with these findings in that we detected increased expression of several stress-response genes in the heads of 35-day-old flies in B:D. These included *cnc* gene, the fly homolog of the transcription factor *Nrf2*, which was also shown to be upregulated in response to blue light in murine retinal pigment epithelium cells.^35^ Importantly, expression of stress-response genes was not elevated in young flies in B:D compared with D:D, which is consistent with our data showing that mortality-inducing stress requires multiple cycles of blue light and is age-dependent. In summary, our data suggest that blue light needs to be added to a range of environmental stressors that become increasingly harmful with repetitive exposure.

Flies are used extensively to understand the mechanisms of aging in laboratories across the world, but the specifics of light conditions in terms of intensity and spectral composition are usually not provided. Our study suggests that the light used in fly facilities may critically affect experimental outcomes and should be reported in aging studies to facilitate the consistency of the results coming from different labs. Our discovery that lifetime exposure to artificial light may cause extra-retinal damage and reduce longevity in a complex model organism provides a novel opportunity to understand the molecular mechanisms of the increasingly evident harmful side of light.

## Methods

### Fly maintenance and genotypes

*Drosophila melanogaster* was maintained on diet containing yeast (35 g/l), cornmeal (50 g/l), and molasses (5%) at 25 ± 1 °C. The genotypes used in this study are described in Supplementary Table 1. Flies used in the experiments were mated and separated by sex when 1–2 days old. Fly colonies were reared in cycles of 12 h of fluorescent light alternating with 12 h of darkness (L:D). Experimental adult flies were maintained in constant darkness or daily cycles of 12-h light from specified light sources.

### Light treatments

Light emitted from different sources was measured at the level where flies were kept by using an SQ-120: Electric Calibration Quantum Sensor (Apogee) and expressed as photon flux density (PFD). The spectrum of each light source was measured with a P100-2-VIS-NIR, optical fiber C (Ocean Optics). During the light phase of the standard L:D cycle in the fly room, flies were exposed to white fluorescent light with an average PFD of 25 µmol m^−2^ s^−1^. In addition, all light sources used in specific experiments are described in Supplementary Table 2. Control flies were kept in constant darkness throughout their adult lifespan and were handled under red light.

### Longevity and behavioral testing

For each genotype and light condition, lifespan was measured by using at least 100 males or 100 females held in groups of 25 in narrow fly vials (Genesee Scientific) with mortality recorded and fresh diet provided every 2–3 days. Mortality curves were statistically analyzed by using the Log-rank test in GraphPad Prism 6. As a behavioral aging biomarker, we tested climbing ability by using the RING assay as described.^36^ Briefly, for each group tested, three vials (without diet), each containing 25 flies, were tapped down (groups being compared were tapped simultaneously) to bring all flies to the bottom of each vial, initiating a rapid negative geotaxis response. Fly upward movement was video recorded, and images were captured 4 s after tapping. These images were analyzed by using NIH ImageJ software to calculate the flies’ average climbing height in each vial. Statistical significance between groups was determined with unpaired *t* tests by using GraphPad Prism 6. To assess locomotor activity, adult males were held individually in glass tubes placed in *Drosophila* Activity Monitors DAM2 or DAM5 (Trikinetics), and activity counts were measured every 15 min. Flies were monitored for five 24-h cycles of L:D or B:D, followed by five 24-h intervals of D:D. Activity data were analyzed by using ClockLab version 2.72 (Actimetrics). Flies were deemed rhythmic if their activity during D:D resulted in an ~24-h periodogram amplitude peak breaking the 99% confidence line, and a Fast Fourier Transform power of 0.04 or above.

### Photoreceptors and brain health

To assess retinal degeneration, we quantitatively determined photoreceptor cell survival on paraffin cross sections of the eye by counting the number of rhabdomeres. The severe disorganization of the ommatidia in some of the conditions made it difficult to identify which of the rhabdomeres belonged to an ommatidia, and we therefore did not count rhabdomeres per ommatidia. Instead, we counted the number of identifiable rhabdomeres in an area of 160 × 160 pixels in images taken at the same magnification (×40) and resolution (1920 × 1440 pixels). To ensure that measurements were done at similar level of the eye, we used images where the anterior–posterior diameter was about 864 pixels and placed the area to be counted in the middle of the image. To quantify light-induced neurodegeneration in the brain, we measured the average area of all vacuoles seen on sections of the brain as described previously.^37^ Analyses were done double-blind, and statistical significance determined with unpaired t tests by using GraphPad Prism 6.

### RNA extraction and qRT-PCR

Frozen heads were separated from bodies by vortexing tubes in liquid nitrogen and with stainless-steel sieves with mesh- opening sizes of 710 and 425 µm. Each sample of 50 heads was homogenized in TRIzol (Thermo Fisher) with a Kontes handheld motorized pestle. RNA was extracted according to the manufacturer’s instructions, and samples were treated with rDNAse I (Takara) followed by phenol/chloroform extraction. RNA was precipitated with ethanol and sodium acetate. cDNA was synthesized from 1 µg of total RNA with the Maxima First Strand cDNA Synthesis Kit (Thermo Fisher). Quantitative real-time polymerase chain reaction (PCR) was performed with Power SYBR Green PCR Master Mix (Thermo Fisher) on a StepOnePlus Real-Time PCR System (Applied Biosystems). Relative expression of genes of interest was calculated by using *DCP2* as the reference gene and 2^−ΔΔCT^ data analysis. All primers (Integrated DNA Technologies) were verified to have >90% efficiency; sequences can be found in Supplementary Table 3.

## Supplementary information

Supplementary Material

Reproducibility checklist

## Data Availability

All data generated or analyzed during this study are included in this published article (and its supplementary information files). Any additional material is available from the corresponding author.
