# Supplementary material for: Daily blue-light exposure shortens lifespan and causes brain neurodegeneration in Drosophila
Source: NPJ Aging Mech Dis. 2019 Oct 17;5:8. doi: 10.1038/s41514-019-0038-6 (PMC6797782; doi:10.1038/s41514-019-0038-6)
Supplement: Supplementary file 1 — Supplementary Material [file 41514_2019_38_MOESM1_ESM.pdf]

SUPPLEMENTARY MATERIAL

Nash et al., Daily blue-light exposure shortens lifespan and causes brain neurodegeneration in *Drosophila*.

LIST OF SUPPLEMENTARY MATERIAL

Supplementary Table 1: Fly stocks used in this study.

Supplementary Table 2: Light sources used in this study.

Supplementary Table 3: Primers used in this study.

SUPPLEMENTARY FIGURES

Supplementary Fig 1. Spectral characteristics of light used in this study.

Supplementary Fig 2. Young *w* males under B:D show increased locomotor activity compared to those in L:D.

Supplementary Fig 3. Clock mutants (per^{01}) had similarly reduced median lifespan to w^{1118} controls in B:D compared to D:D.

Supplementary Fig 4. Median lifespan of *sine oculis* (so^{1}) mutants (which lack the retina and ocelli) was significantly reduced in B:D compared to D:D.

Supplementary Fig 5. Genetic manipulation of *cry* or *Rh7* expression does not alter lifespan of flies in B:D versus D:D compared to their respective controls.

Supplementary Table 1. Fly stocks used in this study.

**Stock**	**Genotype**	**Source**	**Reference**
CS	Canton S	Paul Hardin	
w^{1118} (iso31)	*w*^{1118};;	BDSC stock #5905	[1]
eya^{2}	; *eya*^{2} ;	BDSC stock #2285	[2]
so^{1}	; *so*^{1};	Doris Kretzschmar	[3]
cry^{02}	*w*^{1118} ;; *cry*^{02}	Jeff Hall	[4]
cry-GFP*	*w*^{1118}; *cry-GFP;cry*^{02}	Paul Hardin	[5]
tim-Gal4	*w*^{1118}; {*tim*-Gal4};	Jeff Hall	[6]
UAS-*cry*	*w*^{1118}; {UAS-*cry*-24};	Patrick Emery	[7]
UAS-*cry*^{b}	*w*^{1118}; {UAS-*cry*^{b}-31};	Patrick Emery	[7]
ninaE^{7}	;; *ninaE*^{7}	BDSC stock #2103	[8]
ninaE^{8}	*w*^{1118} ;; *ninaE*^{8}	BDSC stock #2001	[8]
per^{01}	*w*^{1118} *per*^{01} ;;	Jeff Hall	[9]
Tub-GS	{αTub84B-Switch}10	Subhash Katewa	[10]
Rh7^{1}	*w*^{1118}; TI{TI}*Rh7*^{1};	BDSC stock #76022	[11]
UAS-*Rh7***	*w*^{1118};*P{UAS-Rh7.N*}	BDSC stock #76027	[11]

*We exchanged the *cry*^{03} allele, originally in this stock, with *cry*^{02}.

**We removed *Rh7*^{1} allele that was present in this stock.

Supplementary Table 2. Light sources used in this study.

**Referred to as**	**Light source**	**Mfr. and model no.**	**Peak wavelength(s)**	**Photon flux density**
L:D	White fluorescent tubes (cool white, 4100K)	Philips F96T12/CW/VHO/EW	405 nm, 437 nm, 546 nm, 577 nm	20-30 $\mu mol \cdot m^{-2} \cdot s^{-1}$
B:D	Blue LED	MarsAqua Dimmable 165W LED Aquarium Light (Channel 1)	460 nm	20-30 $\mu mol \cdot m^{-2} \cdot s^{-1}$
B+O:D	Blue LED + orange LED	NovoLux LED blue 40 Orange LED Masterled	460 nm, 600 nm	20-30 $\mu mol \cdot m^{-2} \cdot s^{-1}$
W-B:D	White LEDs with yellow long-pass filter	MarsAqua Dimmable 165W LED Aquarium Light (Channel 2); Rosco color filter #4590 CalColor 90 Yellow	584 nm	20-30 $\mu mol \cdot m^{-2} \cdot s^{-1}$

Supplementary Table 3. Primers used in this study.

**Gene Name**	**Symbol**	**FlyBase ID**	**Primers (5' – 3')**
Decapping protein 2	*DCP2*	FBgn0036534	Fwd: CCAAGGGCAAGATCAATGAG<br>Rev: GCATCGATTAGGTCCGTGAT
Glutamate-cysteine ligase catalytic subunit	*Gclc*	FBgn0040319	Fwd: ATGACGAGGAGAATGAGCTG<br>Rev: CCATGGACTGCAAATAGCTG
Glutathione S transferase O1	*GstO1*	FBgn0035907	Fwd: AGCTGTATTCGATGCGCTTT<br>Rev: GGTTTGTCGCGAAGATTGAT
Thioredoxin reductase-1	*Trxr-1*	FBgn0020653	Fwd: AAAAGCAGGATGATGGCAAG<br>Rev: CCACAGAACGGTGTCGTAAA
cap-n-collar	*cncC*<br>(isoform C)	FBgn0262975	Fwd: TCGGAGATGACGAGGAGGAGAGT<br>Rev: GCATTGATGATCGCCTCCTGGT
Glutathione S transferase D1	*GstD1*	FBgn0001149	Fwd: TGAAGCCGGAGTTCCTGAAGA<br>Rev: TGTCGAAGTACAGGCGCTGAT
Glutathione S transferase D2	*GstD2*	FBgn0010038	Fwd: ACATTGCCATCCTGTCCACTG<br>Rev: TCCTGGAGTCACCTTCTTGGC
Heat shock protein 23	*Hsp23*	FBgn0001224	Fwd: GTGTCGAAAATCGGAAAGGA<br>Rev: ACGGAGTTGTCCTGCACTTT
Heat shock protein 68	*Hsp68*	FBgn0001230	Fwd: CCATCATGACCAAGATGCAC<br>Rev: ACGGTGGGACCCTTATAACC
Heat shock protein 70Aa, Ab, Ba, Bb, Bbb, Bc	*Hsp70Aa, Ab, Ba, Bb, Bbb, Bc*	FBgn0013275,<br>FBgn0013276,<br>FBgn0013277,<br>FBgn0013278,<br>FBgn0051354,<br>FBgn0013279	(common to all listed Hsp70 genes)<br>Fwd: CCCACTTTCATTGGGAATTG<br>Rev: TAGTCTGCTTGCACGGAATG
Lactate dehydrogenase	*Ldh*	FBgn0001258	Fwd: CGTTTGGTCTGGAGTGAACA<br>Rev: GCAGCTCGTTCCACTTCTCT

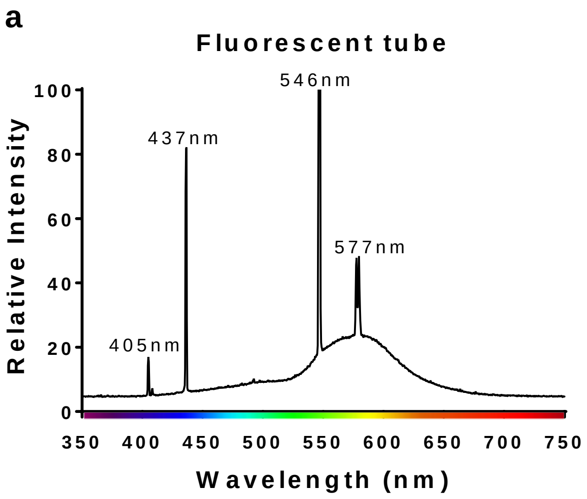

Supplementary Fig 1. Spectral characteristics of light used in this study.

a White fluorescent spectrum (used in L:D conditions). **b** Spectrum of blue LED (used in B:D conditions) with peak wavelength at 460 nm and white LED with blue light blocked by yellow filter (used in W-B:D condition) with peak wavelength at 584nm. Intensity of each light spectrum is normalized to the peak (set as 100%) of the same spectrum.

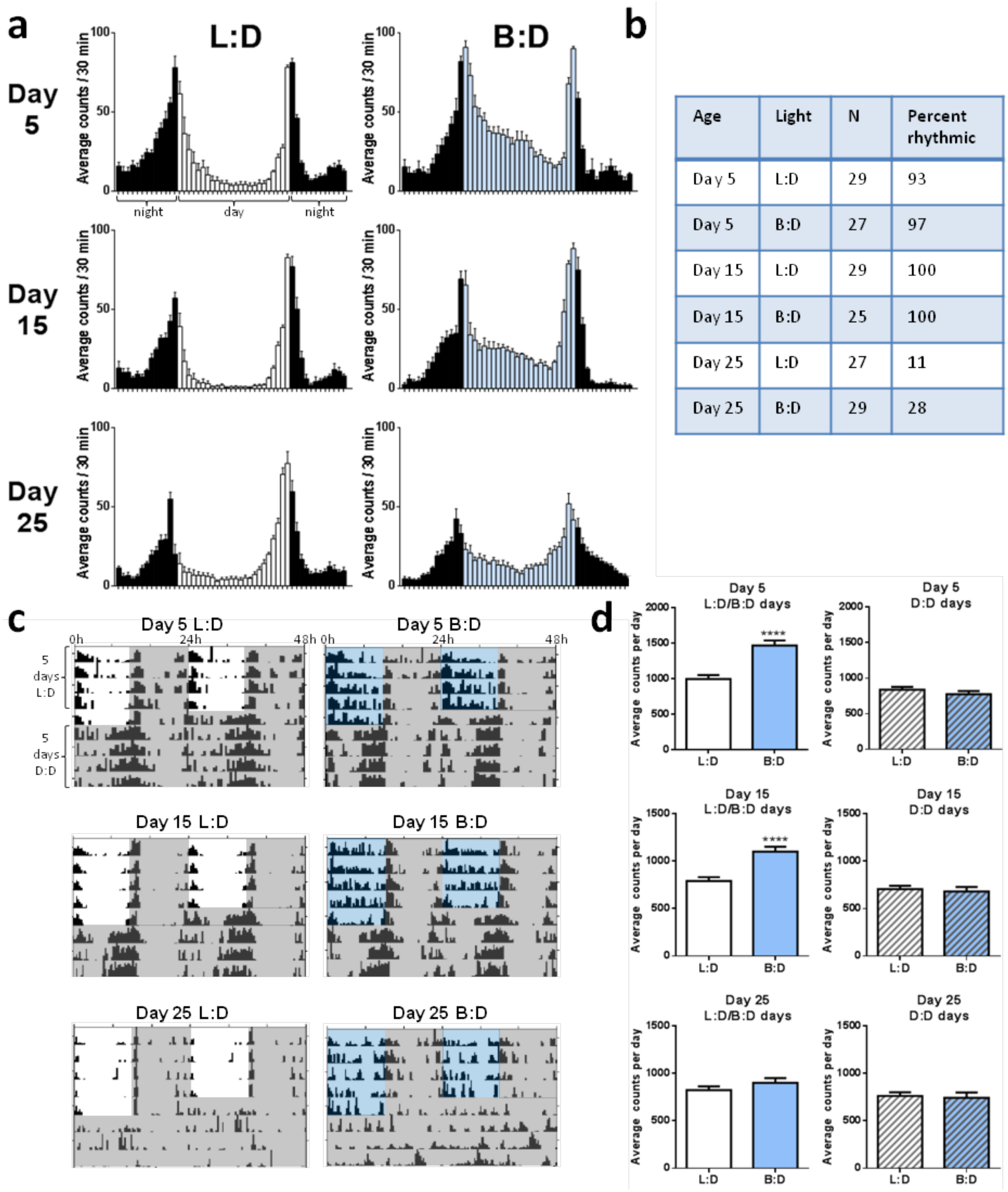

Age	Light	N	Percent rhythmic
Day 5	L:D	29	93
Day 5	B:D	27	97
Day 15	L:D	29	100
Day 15	B:D	25	100
Day 25	L:D	27	11
Day 25	B:D	29	28

Supplementary Fig 2. Young *w* males under B:D show increased locomotor activity compared to those in L:D. **a** Averaged activity counts of all flies in each group, over five consecutive 24 h cycles of L:D or B:D. **b** Summary of experimental details, including percent of rhythmic calculated from D:D days. **c** Representative actograms of individual flies from each group. Gray shaded areas indicate darkness, and white or blue areas indicate white or blue light, respectively. **d** At day 5 and day 15, flies kept in B:D are significantly more active than flies kept in L:D, during the light:dark days (unpaired t-test; ****p<0.0001). No significant difference in activity levels is observed between the same flies which were previously in L:D or B:D, but switched to D:D.

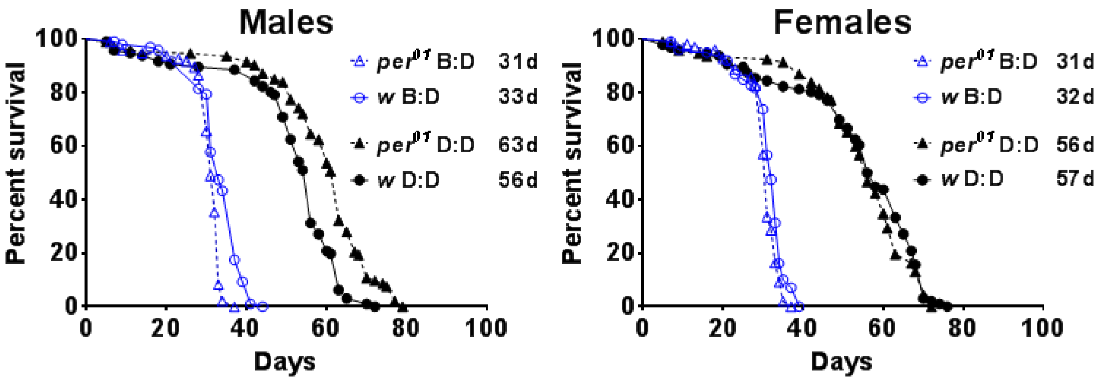

Supplementary Fig 3. Clock mutants ($w^{1118}per^{01}$) had similarly reduced median lifespan to w^{1118} controls in B:D compared to D:D.

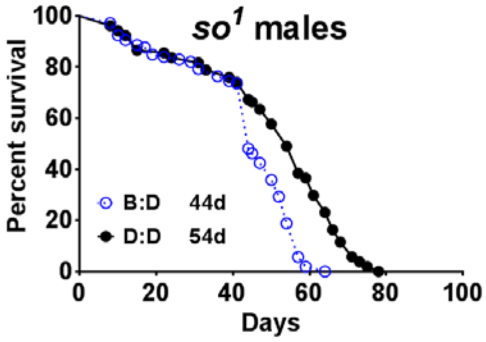

Supplementary Fig 4. Lifespan of *sine oculis* (so^1) mutants, which lack the retina and ocelli, was significantly reduced in B:D compared to D:D. (Log-rank test, $p<0.0001$)

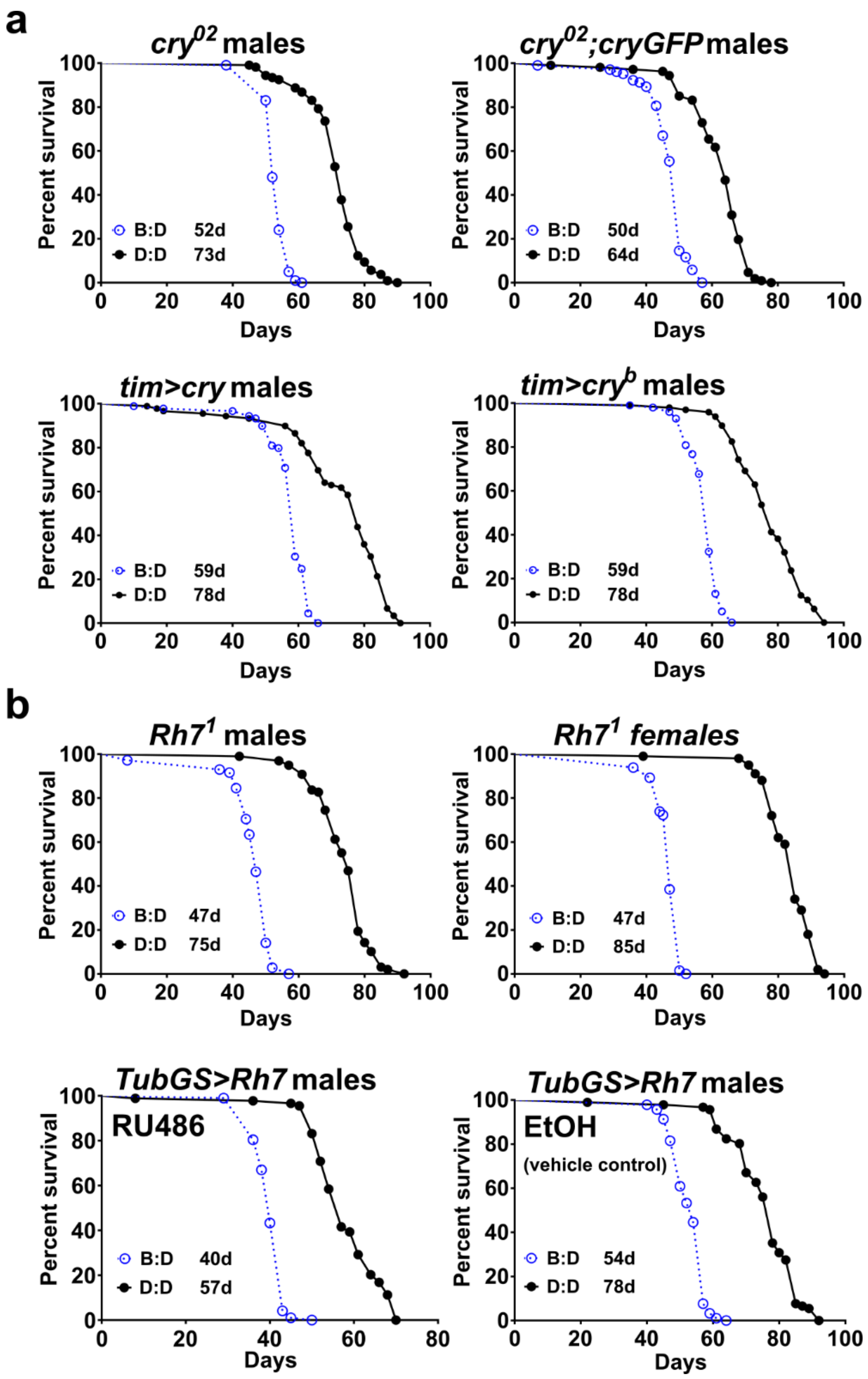

Supplementary Fig 5. Blue light-sensitive proteins CRY or RH7 are not involved in mediating susceptibility to blue light. **a** Mutants not expressing cryptochrome (*cry*^{02}), or flies with elevated expression of this gene via the *tim*-Gal4>UAS-*cry* driver/responder system (left graphs) show similar lifespan differences between B:D and D:D conditions as their respective controls of a *cry* rescue line and *tim*-Gal4>UAS-*cry*^{b} flies overexpressing a nonfunctional version of CRY (right graphs). **b** Mutants lacking Rhodopsin7 (*Rh7*^{1}) and flies overexpressing this gene using the GeneSwitch system induced by the drug RU486 show highly reduced lifespans in B:D compared to D:D. Detailed genotypes are given in Supplementary Table 1.

SUPPLEMENTARY REFERENCES

1. Ryder, E. *et al.* The DrosDel collection: a set of P-element insertions for generating custom chromosomal aberrations in Drosophila melanogaster. *Genetics* **167**, 797-813 (2004).
2. Bonini, N. M., Leiserson, W. M. & Benzer, S. The Eyes Absent Gene - Genetic-Control of Cell-Survival and Differentiation in the Developing Drosophila Eye. *Cell* **72**, 379-395 (1993).
3. Milani, R. Two new eye-shape mutant alleles in Drosophila melanogaster. *Drosophila Information Service* **14**, 52 (1941).
4. Dolezelova, E., Dolezel, D. & Hall, J. C. Rhythm defects caused by newly engineered null mutations in Drosophila's cryptochrome gene. *Genetics* **177**, 329-345. (2007).
5. Agrawal, P. *et al.* Drosophila CRY Entrains Clocks in Body Tissues to Light and Maintains Passive Membrane Properties in a Non-clock Body Tissue Independent of Light. *Curr Biol* **27**, 2431-2441 e2433 (2017).
6. Kaneko, M. & Hall, J. C. Neuroanatomy of cells expressing clock genes in *Drosophila*: transgenic manipulation of the period and timeless genes to mark the perikarya of circadian pacemaker neurons and their projections. *J Comp Neurol* **422**, 66-94 (2000).
7. Busza, A., Emery-Le, M., Rosbash, M. & Emery, P. Roles of the two Drosophila CRYPTOCHROME structural domains in circadian photoreception. *Science* **304**, 1503-1506 (2004).
8. Kumar, J. P. & Ready, D. F. Rhodopsin plays an essential structural role in Drosophila photoreceptor development. *Development* **121**, 4359-4370 (1995).
9. Konopka, R. J. & Benzer, S. Clock mutants of *Drosophila melanogaster*. *Proc. Natl. Acad. Sci. USA* **68**, 2112-2116 (1971).
10. Sykiotis, G. P. & Bohmann, D. Keap1/Nrf2 signaling regulates oxidative stress tolerance and lifespan in Drosophila. *Dev Cell* **14**, 76-85 (2008).
11. Ni, J. D., Baik, L. S., Holmes, T. C. & Montell, C. A rhodopsin in the brain functions in circadian photoentrainment in Drosophila. *Nature* **545**, 340-344 (2017).
